# Consensus on a social return on investment model of physical activity and sport: a Delphi study protocol

**DOI:** 10.3389/fspor.2024.1334805

**Published:** 2024-04-05

**Authors:** I. Nieto, X. Mayo, L. Davies, L. Reece, B. W. Strafford, A. Jimenez

**Affiliations:** ^1^ Sports Science Research Centre, King Juan Carlos University, Madrid, Spain; ^2^ THiNKactive, EuropeActive Research Centre, Brussels, Belgium; ^3^ Sport Policy Unit and Institute of Sport, Manchester Metropolitan University, Manchester, England; ^4^ Sport and Community Capability, Australian Sport Commission, Canberra, ACT, Australia; ^5^ Sport and Physical Activity Research Centre (SPARC) and Academy of Sport and Physical Activity (ASPA), Sheffield Hallam University, Sheffield, England

**Keywords:** social value, physical activity and sports, social return on investment, Delphi, international consensus

## Abstract

**Background:**

Physical activity and sport (PAS) have been related to many health outcomes and social benefits. The main aim of this research is to build a Social Return on Investment (SROI) model of PAS based on experts’ opinion to clarify the domains of impact and how to measure and value them.

**Methods and analysis:**

A Delphi method will be employed with a systematic review on the SROI framework applied to PAS and initial interviews with experts informing the design of the Delphi survey statements. Three iterative rounds of communication with the expert panel will be carried out. Participants will indicate their level of agreement with each statement on a five-point Likert scale. During the second and third iterative rounds, experts will reappraise the statements and will be provided with a summary of the group responses from the panel. A statement will have reached consensus if ≥70% of the panel agree/strongly agree or disagree/strongly disagree after round 3. Finally, group meetings (3–4 experts) will be conducted to ask about the measurement and valuation methods for each domain.

**Discussion:**

The final goal of this project will result in the design of a toolkit for organizations, professionals, and policymakers on how to measure the social benefits of PAS.

## Introduction

During the last decades, life expectancy has increased, with the current average adult life expectancy being 81 years in the European Union (EU) (1). However, these years are characterized by the presence of non-communicable diseases (NCDs), being the main causes of death across Europe, such as cardiovascular diseases, cancers, and neurological disorders (2, 3). Moreover, mental health disorders are also highly prevalent in Europe. In 2019, 7.2% of people in the EU suffered from depression (4) and, during the COVID-19 pandemic, depression among young people more than doubled (1). Given this situation, it is important to promote activities that aim to improve and prevent physical and mental health problems.

Physical inactivity is a major modifiable risk factor for NCDs and mental health conditions (5). However, the relevance of physical inactivity has not been recognised by policymakers until recently. Before 2010, policy documents related to physical activity did not consider the topic as independent but as part of health-related behaviours or together with nutrition (6). It was only in 2013 when the *Vienna Declaration on Nutrition and NCDs in the context of Health 2020 recognised the need to* develop an independent strategy to promote physical activity in the European Region (7). This lack of recognition was evident during the COVID-19 pandemic, when there was a delay in plans for re-opening the resources of the PAS sector, contributing to the “physical inactivity pandemic” (8). In 2022, 45% of EU adults reported that they never exercise or play sport and only 6% reported to exercise or play sport at least five times per week (9). If the minimum requirement of at least 150 min of moderate-intensity exercise per week was met, 10, 331 premature deaths (30–70 years) in a year and 11.5 million NCDs over the next 29 years could be avoided, and 7.7 billion euros per year could be saved in health care expenditure across the 27 EU countries (2). Therefore, it is highly important that policymakers and the PAS industry understand the relevance to promote physical activity in the population.

During the last ten years, the Social Return on Investment (SROI) modelling has been used with this aim. The SROI framework was developed by the Roberts Enterprise Development Fund in 1996 in the US (10) to measure value, that is, relevant social, environmental, and economic change for the population using monetary proxies to represent that change (11). It is considered a comprehensive framework that goes beyond a microeconomic vision (12) and that results in a ratio of monetized social value, for example, a ratio of 3:1 indicates that an investment of 1 euro delivers 3 euros of social value. In order to reach this ratio, an impact map is developed with information on the inputs, outputs, and outcomes of the model (11). *Inputs* are the contribution of the stakeholders for the activity to develop. Financial inputs (e.g., money) are easier to establish than non-monetised inputs (e.g., time), but both need to translate into a financial proxy. *Outputs* are a quantitative summary of the activity, for example, number of people involved, or time spent in a program. Finally, *Outcomes* are the final result of the activity and need to be linked to one or more *indicators* that measure whether the outcome occurred and by how much (e.g., an outcome of the intervention might be reduction of social isolation and the indicator whether participants report having more friends) and also need to translate to a *financial proxy*.

The SROI framework has been used globally by public agencies and third sector organisations to evaluate their social impact, i.e., understand their value to society and justify investment (13). Gosselin et al. (14) conducted the first systematic review of SROI studies applied to PAS and found 17 documents published between 2010 and 2018. Authors highlight some weaknesses in the field such as the large heterogeneity in the application of the SROI method, making it hard to compare evaluations. Also, the quality assessment revealed that many documents did not have a clear rationale for the choice of their SROI framework, which might be explained by the lack of standardization of the model. This limitation is also recognized when choosing the valuation method (financial proxies) which, according to the authors, should be triangulated with a panel of experts. Given the potential of the SROI model, it seems important to develop a standardized framework adapted to PAS in order to measure and value the relevance of these activities and help stakeholders justify their investments.

Academics have been called to action in order to develop and standardize the SROI approach (15, 16). With this purpose, a Delphi methodology will be used in the present study. This technique is a scientific method used to generate information on a topic of interest, especially in situations with limited availability of information (17, 18). It consists of an anonymous, multistage communication process based on several survey rounds with a group of experts (19). The Delphi method has been used in different areas of knowledge such as health care [e.g., (20)], medicine [e.g., (21)], education [e.g., (22)], sports [e.g., (23)], and exercise [e.g., (24)].

Thus, the main aim of this study will be to reach international consensus on (1) the definition of social value applied to PAS, (2) the *Outcomes* that can be measured when applying the SROI methodology to a PAS activity or program, and (3) the *indicators* and *financial proxies* to use for each *Outcome*. The final result of the research will be a comprehensive model to guide any stakeholder interested in the application of the SROI to PAS.

## Methods and analysis

Three main phases will being developed, following Beiderbeck et al.'s (2021) recommendations. Also, the recommendations for the Conducting and REporting of DElphi Studies (CREDES) (25) will be used to ensure transparency and robustness. Details on the phases and recommendations can be found in the Appendix.

### Phase 1. Preparing

The initial conceptualization of the study will be established in a working team meeting, followed by the start of a systematic review on the SROI framework applied to PAS (OFS Registries osf.io/sx9cn). The main goals of this systematic review will be: (1) establishing the *Outcome* categories of social impact within the SROI model applied to PAS, and (2) how to measure and value them, based on previous scientific literature and technical reports. This systematic review will complement the last one in the field (14) by updating the search and by adding information of the *indicators* and *financial proxies* used by each study to measure and value *Outcomes*. This desk research will be conducted in parallel with two creative workshops. The first workshop will take place in Sheffield Hallam University (SHU) with a team formed by an expert in the SROI model (Prof. Larissa Davies), an expert in the Delphi methodology (Dr. Ben Strafford), a health economist at the World Health Organization (Dr. Andreia Costa Santos), experts in the field of physical activity and sports (Dr. Xian Mayo and Prof. Alfonso Jiménez), and an expert in data analysis and psychology (Dr. Inés Nieto). The meeting goals will be to (1) discuss the systematic review progress, (2) agree on the Delphi design, steps, and timeline, (3) establish the framework and questions to conduct the initial interviews, and (4) draft the list of the initial panel of experts. Experts will be defined as active researchers in the field or practical users of the SROI model applied to PAS. Specifically, the following inclusion criteria will be employed to be included as an expert panel member: (1) speak English, and (2) have been an expert in the SROI model for at least 2 years, or have been an expert in the social value domains (health, crime, education, well-being, environment, economy) for at least 3 years, or have been an end user (policy makers and industry) for at least 3 years.

The working team will meet twice after the first workshop to review the process and prepare the second creative workshop. The second workshop will take place at WHO headquarters to get feedback from the design of the project and determine the final statements to include in the Delphi survey. The team will be formed by the same experts present during the first workshop and Dr. Peter Taylor (Emeritus Professor of Applied Sports Economics Sheffield Hallam University), Dr. Lindsey Reece (Director Australian Sports Commission), Dr. Fiona Bull (Unit Head, Physical Activity Department of Health Promotion UHC/Healthier Populations Division, WHO), Dr. Juana Willumsen (Technical Officer Physical Activity Unit Department of Health Promotion UHC/Healthier Populations Division, WHO), and Dr Silvano Zanuso (Director of Technogym's Medical & Visiting Professor at Edith Cowan University).

### Phase 2. Conducting

Phase 2 will start with the pilot of the Delphi survey in *Qualtrics.* This pilot will involve the completion of the survey by the working team, as well as by some of the external advisors from the previous workshops in order to avoid conflicts of interest. The pilot will be followed by the iterative rounds of communications with the final expert panel. This phase will last one month, with three survey rounds separated by 2 weeks each and around 15–20 experts per *Outcome* category. Two reminders will be sent via email to members of the panel between the rounds. Participants will need to indicate their level of agreement with different statements on a five-point Likert scale (strongly agree, agree, disagree, strongly disagree, don't know). These statements will be related to the definition of social value and to the *Outcome* categories that can be measured in relation to the social impact of PAS. The statements related to the social value definition will change from the first to the second round. In the first round, experts will be asked about the components that should be present in the definition of social value (e.g., individual vs. societal level, monetisation). In the second and third rounds, experts will be given a definition created from the answers in the first round in order to show their level of agreement. The statements related to the *Outcome* categories will be the same in the three rounds. In all cases, as it is standard in the Delphi methodology (26), during the second and third iterative rounds experts will be provided with a summary of the group responses from the panel, i.e., the percentage of agreement with the different statements. After the analysis of the three rounds, groups meetings will be conducted with 3–4 experts in each of the *Outcomes* agreed in the survey. These meetings will aim to find consensus on the measurement and valuation methods that should be used in each case.

### Phase 3. Analysing

The initial interviews will be analysed using a reflexive thematic analysis. The reflexive analysis will involve both deductive and inductive coding to identify higher and lower order themes (27). The deductive approach is based on the use of a pre-determined framework to guide the analysis, while the inductive approach is based on the analysis of the data without a previous structure (27, 28). First, a deductive analysis will be used to organise the open-ended questions into dimensions based on the SROI framework structure (Concept of Social Value, *Outcomes*, Measurement, and Valuation) and into higher order themes based on *Outcome* categories (health, crime, education, subjective wellbeing) from previous SROI studies applied to PAS [e.g., (29)]. Then, the inductive approach will be used to refine higher order themes and create lower order themes in relation to the research question. This analysis will be conducted by the lead author and will consist of reading the interviews transcript several times to identify initial codes (i.e., interesting ideas of each interview and quotes relevant to each code). Then, initial codes will be grouped into lower order themes in relation to the research question and linked to the pre-determined theoretical dimensions. Codes classified in more than one of the themes will be assigned into the one perceived to best “fit”. To ensure objectivity and agreement, the analysis will be completed with an interrater agreement in which another author will independently codify two randomly selected interviews (based on previous literature (29, 30) to agree on the thematic structure. The dimensions, higher-order themes, and lower-order themes will inform, together with the results of the systematic review the development of the statements used in the Delphi survey.

The Delphi survey will be analysed considering the percentage of agreement of the panel of experts with the different statements. A statement will be considered to have consensus if ≥70% of the panel agree/strongly agree or disagree/strongly disagree. This criterion will be used to select the components of the social value definition in the first round and the definition in the second and third rounds. Only if this level of agreement is found in the final definition of the third round, it will be considered that there is consensus on what social value means. Moreover, this criterion will be used to include or exclude the *Outcome* categories that should be measured as part of the social impact of PAS. If an *Outcome* category does not reach consensus, it will be excluded from the final model. The items where consensus is not reached will be reported and discussed given that they could provide relevant information for the area (31). Stability of consensus will also be calculated and considered reached if the between round group responses (between rounds 2 and 3) varied by ≤10% (32). The selected *Outcome* categories will guide the recruitment of experts to conduct small group meetings for the last part of the process (i.e., discuss about the best measurement tools and valuation methods to use in each of the areas of expertise to be able to reach the SROI ratio).

The final model will be built based on the consensus obtained from the Delphi survey and the inputs from the group meetings. This model will provide a structure with all the available *Outcomes* to include in a SROI evaluating PAS and the different measurement tools and valuation methods to use for each *Outcome*. This structure will be translated to a practical toolkit designed as a decision-making tree in which the person/organisation interested in the application of the model will need to choose options based on context-specific characteristics, such as the scope of application (local, regional, national, etc.) or the availability of resources (personnel to conduct and analyse surveys, money to measure PAS with gadgets, etc.). This toolkit also aims to provide guidelines on how to deal with important details of the SROI technique (e.g., intermediate outcomes to avoid double counting). The final toolkit will be supervised by all the members of the working team and the participants of the initial workshops, which include SROI experts and external advisors.

## Discussion

Given the potential capacity of the SROI model to be used as a justification for stakeholders to further invest in PAS and the lack of standardization in its application, the aim of the present study will be to reach international consensus on this methodology. Specifically, experts around the world will be asked to give their opinion to build a definition of social value, to select the *Outcomes* that PAS have an impact on, and to find the indicators and financial proxies to measure and value each *Outcome*.

Although social impact measurement has been widely used, “social impact” or “social value” in the recent literature has been misunderstood as the impact on social indicators instead of the impact to society (33). Therefore, it is important to clearly establish the definition of social value applied to PAS. Regarding the *Outcomes*, Gosselin's et al. (2020) systematic review on SROI applied to PAS found that these activities are associated with many different social impacts, from physical health to social trust. While this highlights the relevance of PAS to society, it does not allow to compare between interventions and does not provide stakeholders with solid arguments to invest. Moreover, negative *Outcomes* of PAS to society have been ignored (14, 15, 34), which might bias the resulting ratios and decrease the credibility of the method. The Delphi survey in this study will include statements about the negative outcomes measured in previous studies (as shown in the systematic review) and according to experts suggestions during the initial interviews. It is important that the resulting model of the present project shows a realistic representation of the return to society (positive and negative) from PAS activities.

One of the reasons why the SROI mdoel is promising but lacks wide application is the existence of practical implementation problems (35). First, it deals with concepts which are not always easy to measure, for example, improvement in social relationships or perceived health. Also, studies aiming to calculate the social value of PAS at population level will take the data from secondary sources [e.g., (29)] while studies aiming to calculate the impact of a specific program should monitor their own data [e.g., (36)]. Given these considerations, the operationalization of these variables using experts’ knowledge is much needed.

Moreover, an important part of the SROI model is the monetisation of *Outcomes*. However, it has been noted as one of the main challenges of the method (15, 16, 37) and there is no guidance on how concepts such as self-esteem can be translated to money. There are some tools aiming to help in this process. For example, the HACT social value (38) and the Global Value Exchange database (39) provide information of the values that should be assigned to different social outcomes. However, research applying SROI to PAS have not been consistent in the use of the different valuation methods (14). The SROI framework could benefit from considering the wide range of alternatives (e.g., willingness to pay approach, income compensation approach) and then choose the best option in each case to use it as an international standard. The final phase designed in this study with small group meetings aim to fill this gap. Three or four specialised experts in each *Outcome* will be recruited to discuss and bring light into the best methods to both measure and value the different social impacts. These groups will also help in the reassurance of the survey results. One of the principles of the SROI methodology is the avoidance of overclaiming, which involves the need to justify all the *Outcomes* included in the analysis. For example, environment is one of the hot topics in European policies (40), but is there enough evidence that PAS can have an impact (positive or negative) on environment? Focus groups will bring knowledge on the scientific evidence about each *Outcome* and will help discard the areas where more research is needed.

Finally, it is important to consider the practical implications of the application of an SROI model. The audience in this project is quite diverse, e.g., experts aiming to influence government/councils, third sector organisations, or researchers in the area. This study will support all of them by giving a structured model agreed by experts and translated to a practical toolkit. Given the variety of potential interested stakeholders, this toolkit will not be static, instead it will be designed as a decision-making tree able to adapt to the context of application.

### Limitations

The present research does not aim to get consensus on the frequency and intensity threshold of physical activity to consider its impact. Although future research could be benefited from determining different levels of impact based on different levels of PAS, this is out of the scope of the present study.

The final framework resulting from the present expert consensus should not be taken as universally applied with no supervision and rationale. The SROI modelling is characterized by engagement with stakeholders and the creation of an impact map that adapts to each project, activity, or intervention (11). The resulting model in this research will serve as a comprehensive guide to take as the starting point when thinking about carrying a SROI study. Then, stakeholders are responsible to select the elements that better adapt to their context.

Sample recruitment might suffer from potential low engagement with the study invitation email and attrition expected in the different rounds of the survey. In order to reach consensus among at least 15 experts per area (24), at least 20 of them will be invited using the snowball sampling technique. Although this technique is appropriate to address the study aim, it can also threaten criteria established to include experts. The issues above will seek to addressed by gaining insights on the panel members background questions at the beginning of the first survey.

Finally, this study aims to get international consensus, i.e., representative of the different parts of the world. However, cultural bias might arise during the process. In order to avoid these biases, the authors will invite at least one expert per continent and will supervise the recruitment procedure in the first round to make sure there is sufficient representation. Moreover, the consensus reached in this study should not be considered the only “correct” answer or judgement (41), but it will serve as an initial guide for the standardization of the SROI model in the area of PAS.

## Conclusions

The final result of the present study is a toolkit aiming to help stakeholders in the implementation of the SROI method. Lack of training, dependency on external consultants, and the lack of public resources to learn and compare outcomes will be partly solved. This toolkit will serve as a guide on the steps, variables, tools, and methods to use depending on the activity to evaluate (e.g., population movement, sports program, PA intervention). This contribution will help understand the importance of an active living and increase its promotion.

## Ethics statement

This study was approved by the Ethic Board of the university (internal registration number 2802202309923) and data will be treated following the Regulation (EU) 2016/679 of the European Parliament and of the Council of 27 April 2016 on the protection of natural persons with regard to the processing of personal data and on the free movement of such data, and repealing Directive 95/46/EC (General Data Protection Regulation). Results will be disseminated via scientific articles, reports, and presentation in scientific congresses.

## Appendix

Phases and steps of the Delphi.

**Table:** Conducting and REporting of DElphi studies (CREDES) guidelines (25).

Rationale for the choice of the Delphi technique	Page
1. Justification. The choice of the Delphi technique as a method of systematically collating expert consultation and building consensus needs to be well justified. When selecting the method to answer a particular research question, it is important to keep in mind its constructivist nature	5
Planning and design	
2. Planning and process. The Delphi technique is a flexible method and can be adjusted to the respective research aims and purposes. Any modifications should be justified by a rationale and be applied systematically and rigorously	6–8
3. Definition of consensus. Unless not reasonable due to the explorative nature of the study, an *a priori* criterion for consensus should be defined. This includes a clear and transparent guide for action on (a) how to proceed with certain items or topics in the next survey round, (b) the required threshold to terminate the Delphi process and (c) procedures to be followed when consensus is (not) reached after one or more iterations	9
Study conduct	
4. Informational input. All material provided to the expert panel at the outset of the project and throughout the Delphi process should be carefully reviewed and piloted in advance in order to examine the effect on experts’ judgements and to prevent bias	7
5. Prevention of bias. Researchers need to take measures to avoid directly or indirectly influencing the experts’ judgements. If one or more members of the research team have a conflict of interest, entrusting an independent researcher with the main coordination of the Delphi study is advisable	7
6. Interpretation and processing of results. Consensus does not necessarily imply the “correct” answer or judgement; (non)consensus and stable disagreement provide informative insights and highlight differences in perspectives concerning the topic in question	10, 12–13
7. External validation. It is recommended to have the final draft of the resulting guidance on best practice in palliative care reviewed and approved by an external board or authority before publication and dissemination	10
Reporting	
8. Purpose and rationale. The purpose of the study should be clearly defined and demonstrate the appropriateness of the use of the Delphi technique as a method to achieve the research aim. A rationale for the choice of the Delphi technique as the most suitable method needs to be provided	5
9. Expert panel. Criteria for the selection of experts and transparent information on recruitment of the expert panel, sociodemographic details including information on expertise regarding the topic in question, (non)response and response rates over the ongoing iterations should be reported	7
10. Description of the methods. The methods employed need to be comprehensible; this includes information on preparatory steps (How was available evidence on the topic in question synthesised?), piloting of material and survey instruments, design of the survey instrument(s), the number and design of survey rounds, methods of data analysis, processing and synthesis of experts’ responses to inform the subsequent survey round and methodological decisions taken by the research team throughout the process	6–10
11. Procedure. Flow chart to illustrate the stages of the Delphi process, including a preparatory phase, the actual “Delphi rounds”, interim steps of data processing and analysis, and concluding steps	To include in the final study
12. Definition and attainment of consensus. It needs to be comprehensible to the reader how consensus was achieved throughout the process, including strategies to deal with non-consensus	9
13. Results. Reporting of results for each round separately is highly advisable in order to make the evolving of consensus over the rounds transparent. This includes figures showing the average group response, changes between rounds, as well as any modifications of the survey instrument such as deletion, addition or modification of survey items based on previous rounds	To include in the final study
14. Discussion of limitations. Reporting should include a critical reflection of potential limitations and their impact of the resulting guidance	12–13
15. Adequacy of conclusions. The conclusions should adequately reflect the outcomes of the Delphi study with a view to the scope and applicability of the resulting practice guidance	13
16. Publication and dissemination. The resulting guidance on good practice should be clearly identifiable from the publication, including recommendations for transfer into practice and implementation. If the publication does not allow for a detailed presentation of either the resulting practice guidance or the methodological features of the applied Delphi technique, or both, reference to a more detailed presentation elsewhere should be made (e.g., availability of the full guideline from the authors or online; publication of a separate paper reporting on methodological details and particularities of the process (e.g., persistent disagreement and controversy on certain issues). A dissemination plan should include endorsement of the guidance by stakeholders to facilitate implementation	To include in the final study
